# Protective role of 17β-estradiol in alcohol-associated liver fibrosis is mediated by suppression of integrin signaling

**DOI:** 10.1097/HC9.0000000000000428

**Published:** 2024-05-03

**Authors:** Kruti Nataraj, Michael Schonfeld, Adriana Rodriguez, Irina Tikhanovich

**Affiliations:** Department of Internal Medicine, University of Kansas Medical Center, Kansas City, Kansas, USA

## Abstract

**Background:**

Alcohol-associated liver disease is a complex disease regulated by genetic and environmental factors such as diet and sex. The combination of high-fat diet and alcohol consumption has synergistic effects on liver disease progression. Female sex hormones are known to protect females from liver disease induced by high-fat diet. In contrast, they promote alcohol-mediated liver injury. We aimed to define the role of female sex hormones on liver disease induced by a combination of high-fat diet and alcohol.

**Methods:**

Wild-type and protein arginine methyltransferase (Prmt)6 knockout female mice were subjected to gonadectomy (ovariectomy, OVX) or sham surgeries and then fed western diet and alcohol in the drinking water.

**Results:**

We found that female sex hormones protected mice from western diet/alcohol-induced weight gain, liver steatosis, injury, and fibrosis. Our data suggest that these changes are, in part, mediated by estrogen-mediated induction of arginine methyltransferase PRMT6. Liver proteome changes induced by OVX strongly correlated with changes induced by *Prmt6* knockout. Using *Prmt6* knockout mice, we confirmed that OVX-mediated weight gain, steatosis, and injury are PRMT6 dependent, while OVX-induced liver fibrosis is PRMT6 independent. Proteomic and gene expression analyses revealed that estrogen signaling suppressed the expression of several components of the integrin pathway, thus reducing integrin-mediated proinflammatory (*Tnf*, *Il6*) and profibrotic (*Tgfb1, Col1a1*) gene expression independent of PRMT6 levels. Integrin signaling inhibition using Arg-Gly-Asp peptides reduced proinflammatory and profibrotic gene expression in mice, suggesting that integrin suppression by estrogen is protective against fibrosis development.

**Conclusions:**

Taken together, estrogen signaling protects mice from liver disease induced by a combination of alcohol and high-fat diet through upregulation of *Prmt6* and suppression of integrin signaling.

## INTRODUCTION

Alcohol-associated liver disease (ALD) is a complex disease regulated by a combination of genetic and environmental factors. Sex is an important variable, and it is known that both male and female sex hormone signaling regulates liver disease development. In females, there are 3 types of the naturally produced sex hormone, estrogen: 17β-estradiol (E2), estrone, and estriol. 17β-estradiol is the predominant circulating estrogen. It signals through estrogen receptor alpha, estrogen receptor beta, and G-protein–coupled estrogen receptor. These receptors are most found in reproductive organs and in the liver, which is one of the most responsive organs to E2 treatment.

The classic mechanism of E2 action involves E2 binding to estrogen receptor alpha or estrogen receptor beta. Estrogen receptor then binds to estrogen response elements. In the liver, genes regulated by estrogen response element are genes involved in lipid metabolism, fatty acid synthesis, cholesterol metabolism, and glucose homeostasis. In addition, estrogen receptor can regulate gene expression indirectly. For example, estrogen receptor alpha interacts with NF-κB, preventing NF-κB from promoting cytokine gene expression.^1,2^

Estrogens play a key role in maintaining liver homeostasis. Lack of estrogen signaling promotes liver fat accumulation and leads to hepatic insulin resistance. Estrogen deficiency exacerbates steatohepatitis in humans and rodent models. Interestingly, in high-fat and high-cholesterol diet in mice, estrogen treatment improved inflammation, liver injury, and serum cholesterol levels.^1,2^ In experimental studies, E2 administration protected the liver from hepatic fibrosis by downregulation of the hepatic collagen, hyaluronic acid, and alpha Smooth Muscle Actin expression in male and female rats.^3^ Several studies reported the protective role of estrogens in chronic liver disease, and this has been widely accepted and confirmed using gonadectomy in rodent models. However, in a few clinical studies, increased estrogen levels are also implicated in liver disease. This controversy could be due to differences in environmental factors, such as alcohol exposure.

After alcohol exposure, females exhibit injury more quickly than males. Moreover, female livers, when compared with male livers, show increased TLR signaling and NF-κB activity after ethanol treatment,^4,5^ suggesting higher sensitivity of female liver to endotoxin/Lipopolysaccharide. In fact, several studies confirm that estrogen treatment increases the sensitivity of hepatic macrophages to endotoxin.^4,6^ In addition, estrogen signaling regulates the response of female macrophages to other substances, such as oxysterols,^7^ that are crucial for maintaining KC identity and regulating inflammation in the liver.^8^

The interplay between protein arginine methylation and estrogen signaling is well known.^9–12^ protein arginine methyltransferase PRMT)6 is an enzyme that catalyzes the formation of mono and asymmetric dimethylarginine. PRMT6 was reported to methylate histones and nonhistone proteins; however, under normal conditions, it has a narrow range of substrates when compared with other methyltransferases, such as PRMT1. In contrast, in the presence of alcohol when PRMT1 is inhibited,^13–17^ PRMT6 function becomes critical for maintaining protein methylation.^18^ Despite its limited substrate scope, PRMT6 has been reported to regulate a wide range of cellular processes, including cell cycle, cellular senescence, adipocyte differentiation, neuronal function, hematopoietic stem cell differentiation, and many others. Recently, we found that PRMT6 controls liver fibrosis development in alcohol-fed mice by regulating integrin methylation.^18^ *Prmt6* deficiency resulted in a dramatic increase in liver fibrosis development. However, this effect was less pronounced in females.

In this work, we defined the role of female sex hormone signaling in liver disease development induced by a combination of high-fat diet, western diet (WD), and alcohol exposure using a mouse model. We found that female sex hormones protected mice from WD/alcohol-induced liver disease by promoting PRMT6 expression and suppressing integrin gene expression. Loss of female sex hormone signaling resulted in a decrease of PRMT6 expression, and resulting PRMT6-dependent liver proteomic changes promoted liver steatosis development. On the other hand, loss of estrogen signaling resulted in an increase of several integrin gene expressions that promoted inflammation and fibrosis in mice fed WD/alcohol.

## METHODS

### Mice

*Prmt6* knockout (KO) mice were purchased from Jackson labs (Strain #028929, mixed C57BL/6J and C57BL/6N background) and backcrossed for 5 generations with C57BL/6J mice. Prmt6 +/+, Prmt6 +/−, and Prmt6−/− littermates were used for experiments at 6–8 weeks of age.

All mice were housed in a temperature-controlled, specific pathogen-free environment with 12-hour light-dark cycles. All animal handling procedures were approved by the Institutional Animal Care and Use Committee at the University of Kansas Medical Center (Kansas City, KS). The in vivo experiments are reported according to the ARRIVE guidelines. All animal experiments were approved by the KUMC IACUC, as stated in the Methods section. We report strain, age, weight, sex, and detailed procedures as recommended by ARRIVE guidelines.

### Feedings

For the previously described WD alcohol model,^19^ male and female mice were fed ad libitum WD (Research Diets Inc., Cat# D12079B), and alcohol was given ad libitum in water. Mice received progressively increasing amounts of alcohol in water (1%, 3%, 10%, 15%, and 20% for 3 days each). After reaching 20%, mice were then alternated between 20% (4 days) and 10% (3 days) or stayed at 20% as indicated.

### Gonadectomy

Gonadectomy experiments (ovariectomy, OVX) were performed as described.^20^ Females were maintained on a chow diet until 8 weeks of age. At 6 weeks of age, the mice were gonadectomized under isoflurane anesthesia. Ovaries of female mice were removed through an incision just below the rib cage, the muscle layer sutured, and the incision closed with wound clips. In sham-operated control mice, incisions were made and closed as described above. The gonads were briefly manipulated but remained intact.

### Tandem mass tag (TMT) labeling and mass spectrometric analysis

Whole liver extracts were lysed in 20 mM HEPES (pH 8.0), 150 mM NaCl, and 0.5% NP-40, 0.1% SDS. Trypsin digestion and TMT labeling were performed following the manufacturer’s instructions using the TMT-sixplex Mass tagging kit (ThermoFisher Scientific). Briefly, 100 μg of protein per condition were digested with 2.5 µg of trypsin (Promega). After trypsin digestion, TMT label reagent was added. Equal amounts of labeled samples were combined, and the sample was then loaded into a high pH reverse phase spin column previously conditioned following the manufacturer’s instructions (ThermoFisher Scientific). The peptides were eluted in 9 fractions. Eluted samples were injected into the HPLC coupled with the Orbitrap Fusion Lumos spectrometer (ThermoFisher Scientific). For data analysis, all tandem mass spectrometry scans were searched using Protein Discoverer v.2.4 running Sequest HT and a mouse database downloaded from the NCBI NR repository. Protein quantification was done utilizing unique peptides only.

### Cytokine array

Proteome Profiler Mouse Cytokine Array Kit (R&D Systems) detecting 111 mouse cytokines was used according to the manufacturer’s instructions.

### Macrophage isolation

Primary peritoneal macrophages were isolated as described.^21^ Eight- to 10-week-old mice were killed by CO_2_ asphyxiation. Briefly, 10 mL of sterile PBS was injected into the caudal half of the peritoneal cavity using a 25-gage needle (beveled side up), followed by gentle shaking of the entire body for 10 seconds. Saline containing resident peritoneal cells was collected, and cells were plated on uncoated tissue culture plates and incubated for 60 minutes at 37 °C. Nonadherent cells were removed by washing 5 times with warm PBS. Macrophages were maintained in Roswell Park Memorial Institute (RPMI) culture Medium (Invitrogen) containing 10% fetal bovine serum.

### Western blotting

Protein extracts (50 µg) were subjected to 10% SDS-PAGE, electrophoretically transferred to nitrocellulose membranes (Amersham Hybond ECL, GE Healthcare), and blocked in 3% BSA/PBS at RT for 1 hour. Primary antibodies were incubated overnight at manufacturer-recommended concentrations. Immunoblots were detected with the ECL Plus Western Blotting Detection System (Amersham Biosciences, Piscataway, NJ) with the ODYSSEY Fc, Dual-Mode Imaging system (Li-COR).

### Immunohistochemistry

Liver tissue sections (5-μm thick) were prepared from formalin-fixed, paraffin-embedded samples. The quantification of Sirius Red stained sections was performed in a blinded manner. Immunostaining on formalin-fixed sections was performed by deparaffinization and rehydration, followed by antigen retrieval by heating in a pressure cooker (121 °C) for 5 minutes in 10 mM sodium citrate, pH 6.0, as described.[42] Peroxidase activity was blocked by incubation in 3% hydrogen peroxide for 10 minutes. Sections were rinsed three times in PBS/PBS-T (0.1% Tween-20) and incubated in Dako Protein Block (Dako) at room temperature for 1 hour. After removal of blocking solution, slides were placed into a humidified chamber and incubated overnight with a primary antibody, diluted 1:300 in Dako Protein Block at 4 °C. Ag was detected using the SignalStain Boost immunohistochemistry detection reagent (catalog # 8114; Cell Signaling Technology, Beverly, MA), developed with diaminobenzidine (Dako, Carpinteria, CA), counterstained with hematoxylin (Sigma-Aldrich), and mounted.

### RT-PCR

RNA was extracted from livers using the Rneasy Mini Kit (Qiagen). Complementary DNA was generated using the RNA reverse transcription kit (Applied Biosystems, Cat.No 4368814). Quantitative real-time reverse transcription- polymerase chain reaction was performed in a CFX96 real-time system (Bio-Rad) using specific sense and antisense primers combined with iQ SYBR Green Supermix (Bio-Rad) for 40 amplification cycles: 5 seconds at 95 °C, 10 seconds at 57 °C, and 30 seconds at 72 °C. mRNA concentrations were calculated relative to Actb and normalized to the average expression of the control condition.

**Table:** 

Actb fwd	ATGTCACGCACGATTTCCCT
Actb rvs	CGGGACCTGACAGACTACCT
Col1a1-fwd	TGGCCAAGAAGACATCCCTG
Col1a1-rvs	GGGTTTCCACGTCTCACCAT
Tgfb1-fwd	TACGTCAGACATTCGGGAAGC
Tgfb1-rvs	TTTAATCTCTGCAAGCGCAGC
Tnf- fwd	CTGAGACATAGGCACCGCC
Tnf- rvs	CAGAAAGCATGATCCGCGAC
Prmt6 fwd	CACCGGCTCGTTCAAGTAGA
Prmt6 rvs	AAACCTCTGGTGCTGTCCAC
Ilk fwd	GAACGACCTCAATCAGGGGG
Ilk rvs	CATTAATCCGTGCTCCACGC
Itga1 fwd	GTCTGCTTAATTGGTTCCAGGC
Itga1 rvs	AGGTGTACGTGTACGCTGTG
Itga4 fwd	ATCAACTCTGGCATGGGAGC
Itga4 rvs	GACTCCCCAAATCTTGCAGC
Itgax fwd	GCTGGCTATCATCACAGCTATAC
Itgax rvs	TTGCTTCCTCCAACATCTCCTT
Itgb1 fwd	AGCTAATCATCGATGCCTACAACT
Itgb1 rvs	CTCCGTCTGGCAATTTGCTATT
Itgb2 fwd	CCCTCAACGAGATCACCGAG
Itgb2 rvs	GTTGGGACATGGGTTCCTCA

### Vectors

Small hairpin RNA plasmids were from Sigma:

**Table:** 

Cat#	Target
TRCN0000066375	*Itgax*
TRCN0000066043	*Itga4*

### Antibodies

**Table:** 

PRMT6	Santa Cruz	#sc-271744
Integrin α4	Cell Signaling	#4749
COL1A1	Cell Signaling	#91144
αSMA	Cell Signaling	#19245
TGFβ1	Cell Signaling	#3709

### Measurement of liver triglyceride content

Triglycerides (TGs) were measured as described.^22^ Bioprotocol ID: BioProtoc.223.

### Statistics

Student *t* test was used for the comparison of 2 groups, and ANOVA followed by a post-hoc test for 3 or more groups. When data do not show a normal distribution, the Mann-Whitney *U* test for 2 groups or the Kruskal-Wallis test for 3 or more groups was used. Multiplicity adjustment was performed with the Bonferroni multiple comparisons test to control the false discoveries when multiple groups are considered.

## RESULTS

### Female sex hormones protect from liver disease development in alcohol and high-fat diet-fed mice

**FIGURE 1:**
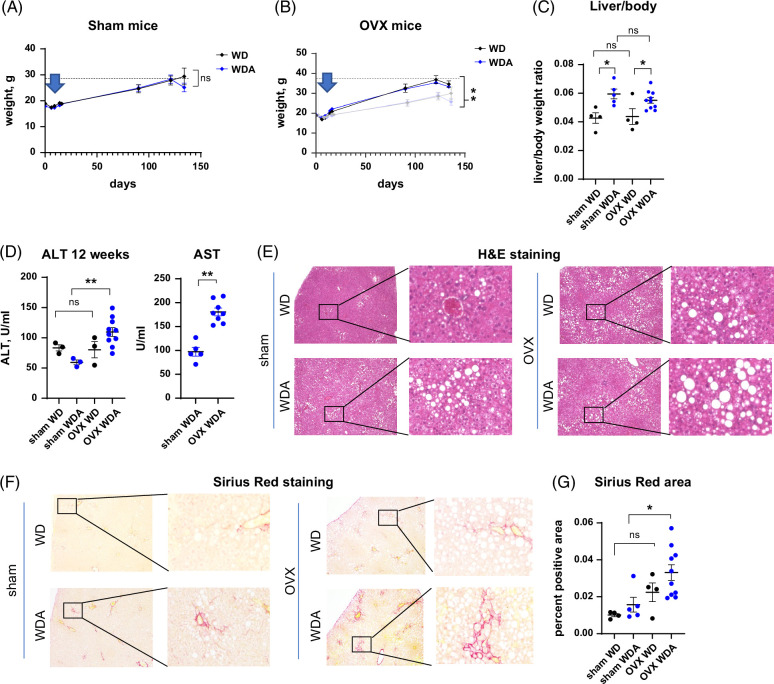
Female sex hormones protect from alcohol and high-fat diet-induced liver injury and fibrosis. Female wild-type mice were subjected to gonadectomy (OVX) or sham surgery and placed on WD control diet or WD with alcohol in the drinking water (alternating 10% and 20%) for 18 weeks. (A and B) Weight change over time in sham and OVX mice fed WD or WDA from 4 groups. **, *p*<0.001 between OVX and sham controls, by linear regression comparison. (C) Liver-to-body weight ratios N=4–10 mice per group. *, *p*<0.05, by one-way ANOVA with Bonferroni multiple comparisons test. (D) Serum ALT at 12 weeks of feeding. N=3–10 mice per group. **, *p*<0.01, by one-way ANOVA. (E). Representative images of H&E staining in mice subjected to sham surgery or gonadectomy (OVX). (F) Representative images of Sirius red staining. (G) Percent positive Sirius Red staining area. N=4–10 mice per group. *, *p*<0.05, by one-way ANOVA with Bonferroni multiple comparisons test. Abbreviations: ALT, alanine aminotransferase; AST, aspartate aminotransferase; H&E, Hematoxylin and eosin; OVX, ovariectomy; WD, western diet; WDA, western diet with alcohol.

To test the role of female sex hormones in high-fat diet and alcohol-induced liver disease, we fed mice either high-fat WD and plain water as a control or western diet with alcohol (WDA) in the drinking water, as described.^18,19^ Female mice underwent gonadectomy (OVX) or sham surgery at 6 weeks of age and were placed on WD control or WDA alcohol diet at 8 weeks of age. After 18 weeks of feeding, we observed that OVX mice gained 60% more weight during WD feeding (Figure 1A, B), and alcohol did not affect the weight gain in agreement with previously described data.^18^ Despite differences in weight gain, in both groups (OVX and sham), alcohol induced a comparable increase in liver-to-body weight ratios (Figure 1C). We found that OVX group fed WD with alcohol had significantly elevated serum alanine aminotransferase (ALT) levels compared to sham controls. In contrast, in the WD-only group, there was no difference in ALT levels (Figure 1D), suggesting that in the presence of high-fat diet, female sex hormones were protected from alcohol-induced liver injury.

Next, we examined histology and liver fibrosis in 4 groups of mice using Hematoxylin and eosin staining (Figure 1E) and Sirius red staining (Figure 1F). We found that alcohol resulted in a mild liver steatosis in sham mice, which was further elevated in OVX mice (Figure 1E). These changes correlated with increased liver fibrosis in OVX mice fed WD with alcohol compared to sham controls (Figure 1F, G). Taken together, alcohol and OVX surgery additively promoted liver injury, steatosis, and fibrosis.

**FIGURE 2:**
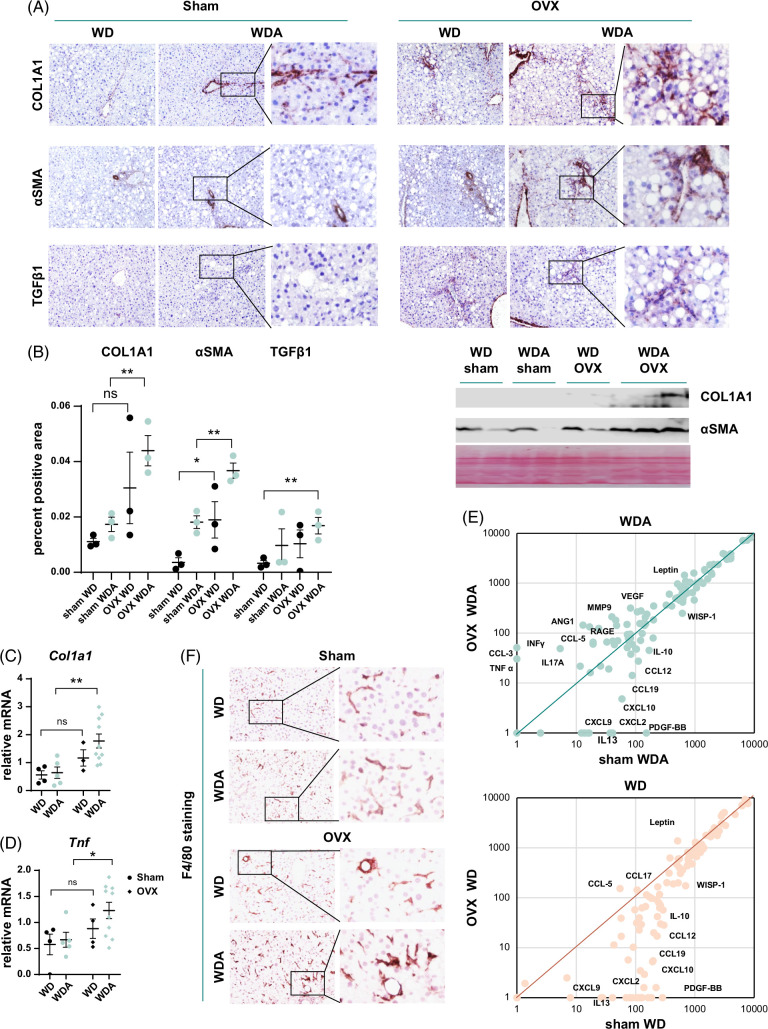
Female sex hormones protect from alcohol and high-fat diet–induced inflammation and fibrosis. Female wild-type (WT) mice were subjected to gonadectomy (OVX) or sham surgery and placed on WD control diet or WD with alcohol in the drinking water (alternating 10% and 20%) for 18 weeks. (A) Representative images of immunohistochemistry staining using COL1A1, αSMA, and TGFβ1 specific antibodies. (B) Percent positive staining area. N=3 mice per group *, *p*<0.05, **, *p*<0.01 by one-way ANOVA with Bonferroni multiple comparisons test. Right. Western blot analysis of COL1A1, αSMA proteins from 4 groups. (C and D) Whole liver mRNA N=4–10 mice per group. *, *p*<0.05, **, *p*<0.01 by one-way ANOVA. (E) Serum cytokine profile at 12 weeks of feeding in wild-type mice. (F) Representative images of F4/80 staining for WT sham and neutralized (OVX) mice of both WD and WDA diets. Abbreviations: α-SMA, alpha smooth muscle actin; CCL, chemokine (C-C motif) ligand; COL1A1, Collagen 1A1; CXCL, Chemokine (C-X-C motif) ligand; MMP9, Matrix metalloproteinase-9; OVX, ovariectomy; PDGF, Platelet-derived growth factor; RAGE, receptor for advanced glycation endproducts; WD, western diet; WDA, western diet with alcohol; WISP-1, WNT1-inducible-signaling pathway protein 1.

Next, we further examined liver fibrosis using immunohistochemistry staining (Figure 2A). We found an increase in Collagen 1A1 (COL1A1), αSMA, and TGFβ1 staining in alcohol-fed OVX mice compared to sham controls (Figure 2A, B), which correlated with increased *Col1a1* mRNA levels (Figure 2C).

Elevated liver fibrosis correlated with an increase in inflammation markers such as *Tnf* (Figure 2D). Since estrogen is known to modulate inflammatory pathways, we examined the circulating cytokine profile in WD-fed and WDA-fed mice using a cytokine array (Figure 2E). We found that in WD-fed mice, OVX resulted in an increase in leptin and a decrease in several chemokines such as CXCL9, CXCL10, and CCL-12 (Figure 2E). In contrast, in the presence of alcohol in addition to the abovementioned changes, OVX promoted an increase in TNFα, IL-17A, CCL-5, RAGE, VEGF, and MMP9, which are known to contribute to liver inflammation and fibrosis. We confirmed that the livers of WDA-fed OVX mice showed higher positive staining for F4/80, and presence of crown-like structures, and inflammatory foci, suggesting that liver inflammation is higher in this group (Figure 2F).

### 17β-estradiol promotes expression of PRMT6 in the liver

**FIGURE 3:**
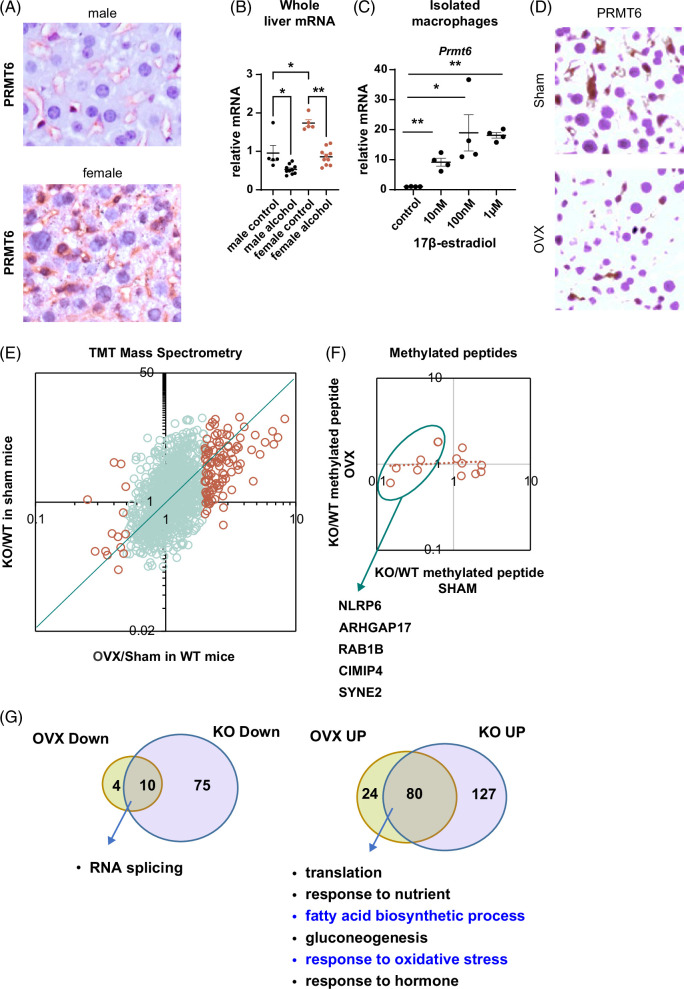
Female sex hormones promote profound proteomic changes in the liver in PRMT6 dependent way. (A–D) Estrogen promotes PRMT6 expression. (A) Representative images of PRMT6 protein staining in livers of male and female mice fed chow diet. (B) Mice (male and female) were fed WD (control) or WD with alcohol in the drinking water (alcohol). Relative *Prmt6* gene expression. N=5–10 mice per group. *, *p*<0.05, **, *p*<0.01 by one-way ANOVA with Bonferroni multiple comparisons test. (C) Relative *Prmt6* gene expression in mouse peritoneal macrophages treated in vitro with 10 nM, 100 nM, or 1 µM 17β-estradiol solution. N=4 independent experiments. *, *p*<0.05, **, *p*<0.01 01 by one-way ANOVA. (D) Representative images of PRMT6 protein staining in mice subjected to sham surgery or gonadectomy (OVX). (E) TMT mass spectrometry analysis of wild type and *Prmt6* knockout mice that were subjected to sham or gonadectomy surgeries and fed WD with alcohol (N=3 per group). The ratio of protein abundance in gonadectomy/sham groups (OVX/Sham) in WT plotted versus ratio of protein abundance in KO/WT sham mice. (F) Fold change in methylated peptide abundances (KO/WT) in mice from gonadectomy (OVX) and Sham groups (G) GO term biological process enrichment analysis in top upregulated and downregulated proteins. Abbreviations: ARHGAP17, Rho GTPase Activating Protein 17; CIMIP4, ciliary microtubule inner protein 4; PRMT6, protein arginine methyltransferase 6; KO, knockout; OVX, ovariectomy; RAB1B, Ras-related protein Rab-1B; SYNE2, Spectrin Repeat Containing Nuclear Envelope Protein 2; TMT, tandem mass tag; WT, wild type.

Previously we found that PRMT6, which primarily is expressed in nonparenchymal cells such as liver macrophages, is a key regulator of liver fibrosis development in both high-fat diet and alcohol-fed mice, and loss of PRMT6 promotes fibrosis and steatosis in liver.^23^ We observed that females have higher levels of PRMT6 protein (Figure 3A) and mRNA (Figure 3B). These differences are likely due to estrogen signaling since treatment of isolated macrophages with 17β-estradiol resulted in a dose-dependent increase in *Prmt6* expression (Figure 3C). In agreement with these data, OVX mice had reduced levels in PRMT6 (Figure 3D).

To evaluate the role of PRMT6 in female sex hormone signaling in the liver, we compared proteomic changes in OVX versus sham mice and proteomic changes in *Prmt6* KO mice compared to wild-type (WT) controls using TMT Mass Spectrometry analysis (Figure 3E).^19,24^ This analysis was performed in alcohol-fed mice only. We found a strong positive correlation between these 2, suggesting that OVX-induced proteomic changes could be mediated by the loss of PRMT6. In addition, we were able to quantify the abundance of several arginine-methylated peptides in these mice (Figure 3F). Five of them (NOD‐like receptor family pyrin domain containing 6, ARHGAP17, RAB1B, CIMIP4, and SYNE2) showed reduced abundance in KO mice compared to WT controls, suggesting that they could be targets of PRMT6 enzymatic activity. We noted that methylation of these proteins was not affected or affected to a lesser extent by PRMT6 KO in OVX mice (Figure 3F), further confirming that PRMT6 activity is reduced in OVX mice. We next analyzed the gene ontology term enrichment in upregulated and downregulated proteins in OVX mice (Figure 3G). We found that upregulated proteins were enriched for proteins involved in fatty acid metabolism and oxidative stress response, suggesting that these changes could contribute to OVX-induced liver injury and steatosis.

### PRMT6 mediates protective effect of female hormone signaling

**FIGURE 4:**
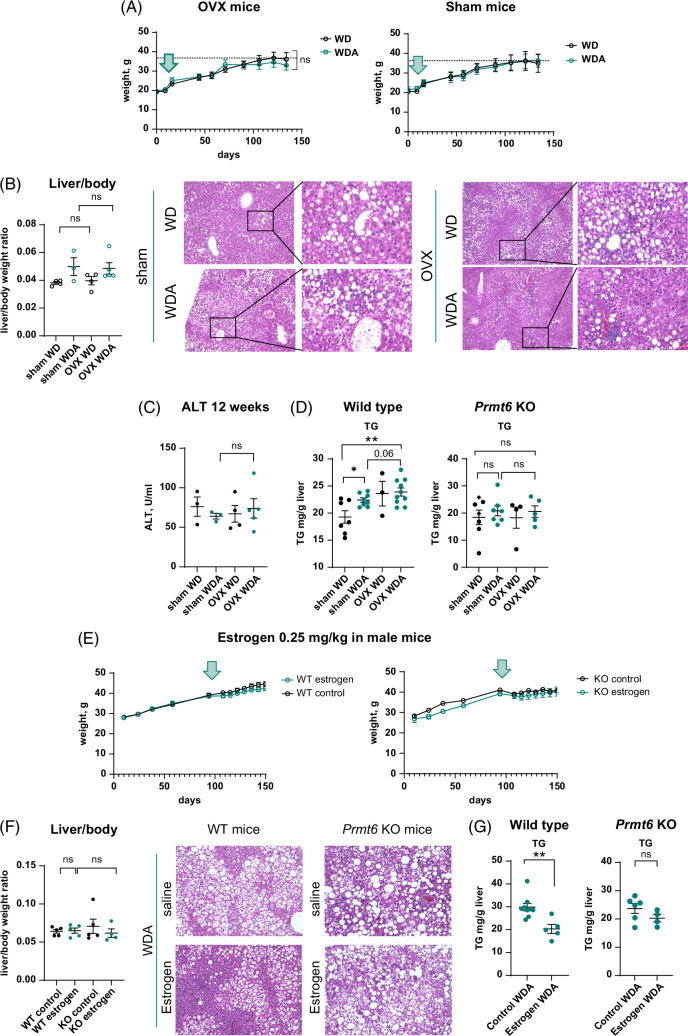
Female sex hormones protect from alcohol and high-fat diet-induced steatosis. (A–D) Female *Prmt6* knockout mice were subjected to gonadectomy (OVX) or sham surgery and placed on WD control diet or WD with alcohol in the drinking water (alternating 10% and 20%) for 18 weeks. (A) Weight change over time in mice from 4 groups. (B) Liver-to-body weight ratios in KO mice, N=3–10 mice per group. Right. Representative images of H&E staining from mice subjected to sham surgery or gonadectomy (OVX) fed WD or WDA diet. (C) Serum ALT at 12 weeks of feeding N=3–5 mice per group. (D) Liver triglyceride measurement in WT and *Prmt6* KO mice. N=3–10 mice per group. *, *p*<0.05, **, *p*<0.01 01 by one-way ANOVA. (E–G) Male mice were fed WD with alcohol in the drinking water for 20 weeks. Starting week 16 mice received twice weekly 0.25 mg/kg of 17β-estradiol in PBS, IP. (E) Weight change over time in WT and *Prmt6* KO mice injected with estrogen or control (saline). Arrow indicates the start of the treatment. (F) Liver-to-body weight ratios in WT and KO mice. Right. Representative images of H&E staining (G) Liver triglyceride measurement in WT and *Prmt6* KO mice. N=4–8 mice per group. **, *p*<0.01 by Student *t* test. Abbreviations: ALT, alanine aminotransferase; KO, knockout; OVX, ovariectomy; TG, triglycerides; WD, western diet; WDA, western diet with alcohol; WT, wild type.

To assess whether PRMT6 mediates the protective effect of female hormone signaling in the liver, we performed OVX or sham surgeries in *Prmt6* KO mice and fed them WD or WD with alcohol for 18 weeks (Figure 4A). We found that in contrast to WT mice, there was no difference in weight gain. All groups gained similar weight, which was comparable to the weight gain of OVX WT mice (Figure 4A compared to Figure 1B). Histologically, we observed that all 4 groups of mice had similar steatosis (Figure 4B) and showed no difference in serum ALT (Figure 4C). To quantitatively assess the liver steatosis phenotype in these mice, we measured liver TG levels (Figure 4D). We found that in WT mice, OVX and alcohol additively increased TG levels. In contrast, in *Prmt6* KO mice, there was no difference between groups (Figure 4D).

To further assess the role of estrogen signaling in liver steatosis in WD and alcohol-fed mice, we treated male mice that were fed WD with alcohol in the drinking water for 20 weeks with exogenous estrogen (17β-estradiol, 0.25 mg/kg twice a week starting week 16). We observed that WT male mice treated with estrogen showed slightly reduced weight gain after the beginning of treatment, which did not reach statistical significance (*p*=0.1). *Prmt6* knockout mice showed no difference in weight gain after estrogen treatment (Figure 4E). We found that estrogen treatment greatly reduced steatosis in male mice (35% reduction in liver TG) without affecting liver-to-body weight ratios (Figure 4F, G). In contrast, in *Prmt6* KO mice, estrogen treatment did not significantly affect liver steatosis (18% reduction, *p*>0.05, Figure 4F, G).

Taken together, estrogen-mediated PRMT6 upregulation protects female mice from WD/alcohol-induced weight gain and liver steatosis.

### Female sex hormones protect from alcohol and WD-induced fibrosis in PRMT6 independent way

**FIGURE 5:**
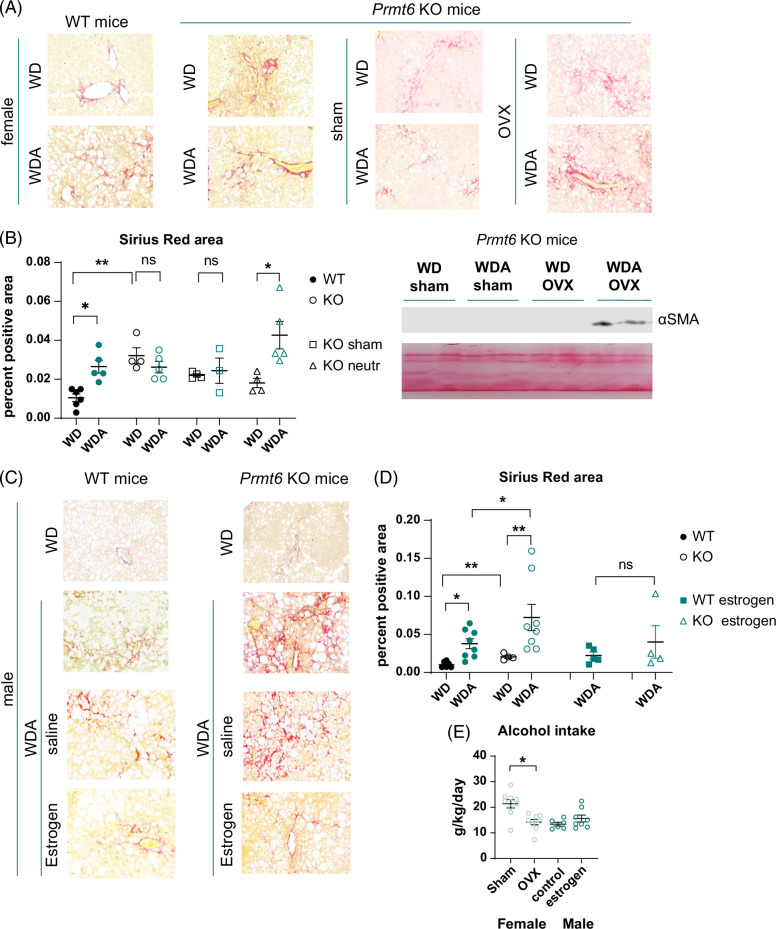
Female sex hormones protect from alcohol and high-fat diet–induced liver injury and fibrosis in a PRMT6-independent way. (A and B) *Prmt6* knockout mice were subjected to gonadectomy (OVX) or sham surgery and placed on WD control diet or WD with alcohol in the drinking water (alternating 10 and 20%) for 18 weeks. (A) Representative images of Sirius red staining. (B) Percent positive Sirius Red staining area. N=3–6 mice per group. *, *p*<0.05, **, *p*<0.01 by one-way ANOVA with Bonferroni multiple comparisons test. Right. Western blot analysis of αSMA protein levels from 4 groups. (C and D) Male mice were fed WD with alcohol in the drinking water for 20 weeks. Starting week 16 mice received twice weekly 0.25 mg/kg of 17β-estradiol in PBS, IP. (C) Representative images of Sirius red staining. (D) Percent positive Sirius Red staining area. N=4–8 mice per group. *, *p*<0.05, **, *p*<0.01 by one-way ANOVA with Bonferroni multiple comparisons test. (E) Alcohol intake in sham control or OVX females (red circles) and control and control (saline) or estrogen-treated male (blue circles) mice. Abbreviations: αSMA, alpha smooth muscle actin; KO, knockout; OVX, ovariectomy; WD, western diet; WDA, western diet with alcohol; WT, wild type.

We next examined the effect of OVX in *Prmt6* KO mice on liver fibrosis by Sirius Red staining (Figure 5A, B). We found that *Prmt6* KO in WD-fed female mice promoted liver fibrosis, and alcohol feeding similarly promoted liver fibrosis in WT females. However, in *Prmt6* KO mice, alcohol did not produce additional increase in liver fibrosis. Similar results were observed in sham *Prmt6* KO mice, which had liver fibrosis levels higher than WT WD controls, but no additional effect of alcohol was observed. In contrast, the OVX mice group showed significantly elevated fibrosis levels after alcohol feeding.

Taken together, in WD-fed WT mice, OVX resulted in a small increase in liver fibrosis (Figures 1 and 2), which is likely due to PRMT6 loss (since the OVX effect is absent in KO WD-fed mice). In contrast, in WD/alcohol-fed females, OVX promoted an additional increase in fibrosis independent of PRMT6.

We next evaluated the effect of estrogen treatment on fibrosis in male mice (Figure 5C, D). In male mice fed WD, *Prmt6* KO resulted in a small increase in liver fibrosis similarly to female mice. In WD/alcohol-fed males, alcohol further increased liver fibrosis in both WT and KO mice, similarly to OVX females but in contrast to female sham controls. Estrogen treatment reduced liver fibrosis in males abolishing the difference between WT and *Prmt6* KO.

Taken together, estrogen suppressed WD/alcohol-induced liver fibrosis development in both male and female mice independent of PRMT6. These effects were not due to altered alcohol intake. We found that OVX females consumed less alcohol than sham controls but had more liver fibrosis. In male mice, we observed the opposite; estrogen-treated mice had slightly higher alcohol consumption (Figure 5E).

**FIGURE 6:**
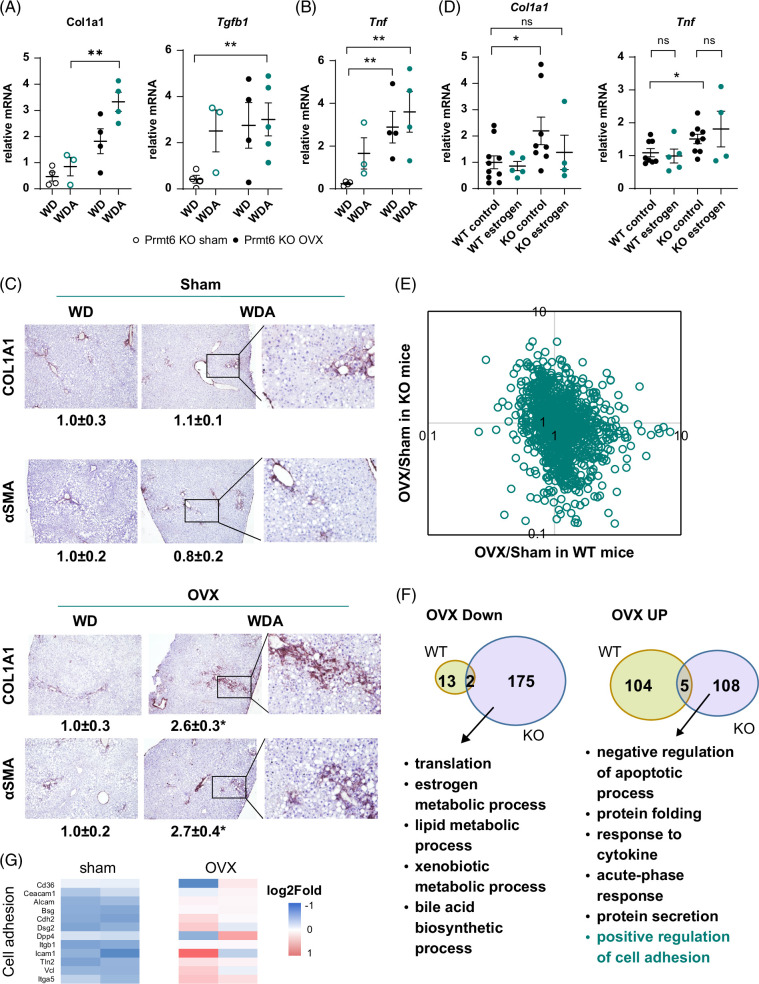
Female sex hormones protect from alcohol and high-fat diet-induced inflammation and fibrosis in *Prmt6* KO mice. (A and B) *Prmt6* knockout mice were subjected to gonadectomy (OVX) or sham surgery and placed on WD control diet or WD with alcohol in the drinking water (alternating 10 and 20%) for 18 weeks. (A and B) Whole liver mRNA N=3–5 mice per group. **, *p*<0.01 by Student *t* test. (C) Representative images of immunohistochemistry staining of liver tissue using COL1A1 or αSMA specific antibodies. N=3. *, *p*<0.05 compared to WD control by student *t* test. (D) Whole liver mRNA for WT and KO male mice injected with estrogen or control, N=5–10 mice per group. *, *p*<0.05 by student *t* test. (E) The ratio of protein abundance in gonadectomy/sham groups (OVX/Sham) in WT and KO mice. (F) GO term biological process enrichment analysis in top upregulated and downregulated proteins. (G) Relative protein abundance of cell adhesion molecules in KO mice subjected to sham (Sham) surgery or gonadectomy (OVX). Abbreviations: αSMA, alpha smooth muscle actin; COL1A1, collagen 1A1; GO, gene ontology; KO, knockout; OVX, ovariectomy; WD, western diet; WDA, western diet with alcohol; WT, wild type.

We further assessed levels of liver fibrosis and inflammation in these mice (Figure 6A–C). We found that increased liver fibrosis in OVX *Prmt6* KO mice fed alcohol correlated with elevated mRNA levels of *Col1a1*, *Tgfb1*, and *Tnf* (Figure 6A, B), similarly to WT OVX mice. We observed that these mice also showed elevated COL1A1 and αSMA staining (Figure 6C). In male mice, WD/alcohol-fed KO mice showed significantly elevated *Col1a1* and *Tnf* mRNA compared to WT controls. Estrogen treatment reduced *Col1a1* levels in KO mice to the level of WT controls; however, *Tnf* levels in KO mice were unchanged (Figure 6D).

### Female sex hormones suppress integrin signaling in the liver

To assess the mechanism of female sex hormone–mediated liver fibrosis regulation, we examined proteomic changes in OVX KO mice compared to sham control (Figure 6E, F). We found that OVX-induced proteomic changes were different in WT and KO mice (Figure 6E). Gene ontology term enrichment analysis of proteins regulated by OVX in KO mice (Figure 6F) showed that proteins upregulated by OVX in KO mice included several molecules involved in cell adhesion, such as integrins (Figure 6G).

**FIGURE 7:**
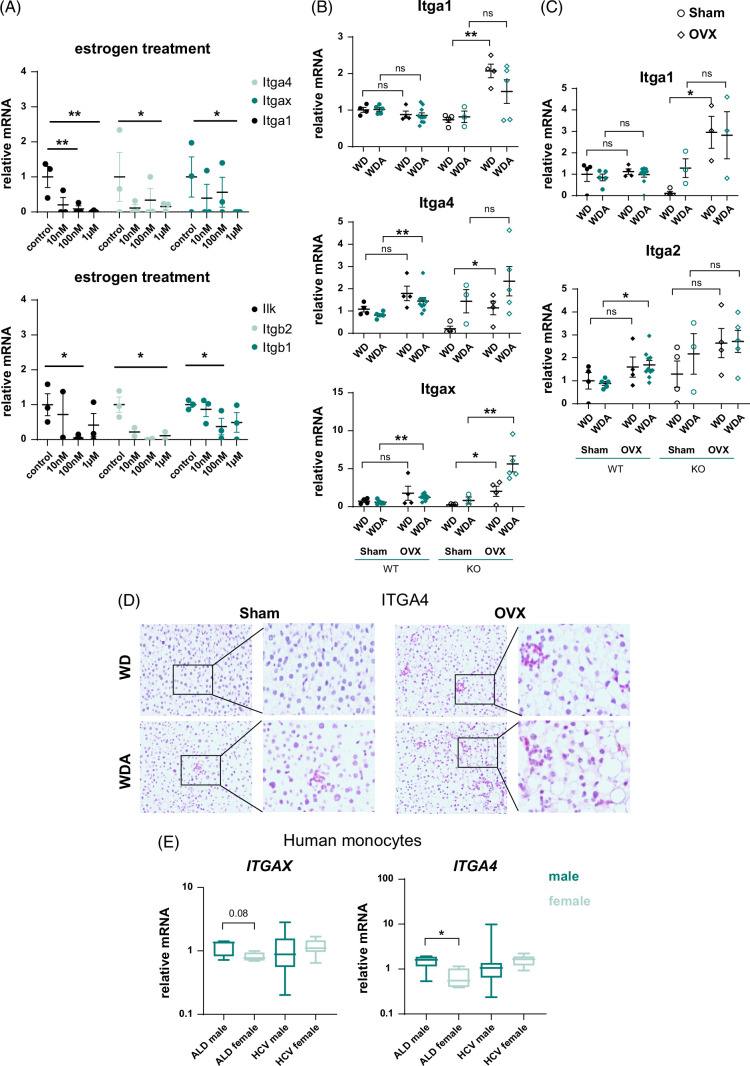
Estrogen suppresses integrin expression. (A) Relative gene expression in mouse peritoneal macrophages treated *in vitro* with 10 nM, 100 nM, or 1 µM 17β-estradiol solution. N=3 independent experiments. *, *p*<0.05, **, *p*<0.01 by paired *t* test. (B and C) Female wild-type and *Prmt6* KO mice were subjected to gonadectomy (OVX) or sham surgery and placed on WD control or WDA alcohol diet for 18 weeks. Whole liver mRNA. N=3–10 mice per group. *, *p*<0.05, **, *p*<0.01 by one-way ANOVA with Bonferroni multiple comparisons test in WT and KO mice separately. (D) Representative images of immunohistochemistry staining in WT female mice using integrin α4 (ITGA4) specific antibody. (E) Relative gene expression in blood monocytes from female and male patients with ALD or HCV *, *p*<0.05 by Student *t* test. Abbreviations: ALD, alcohol-associated liver disease; *Ilk*, integrin-linked kinase; KO, knockout; OVX, ovariectomy; WD, western diet; WDA, western diet with alcohol; WT, wild type.

We next examined whether estrogen and female hormone signaling modulated integrin pathway (Figure 7). We found that in isolated macrophages, estrogen treatment suppressed the expression of several integrin genes, including genes for integrins α1, α4, αx, β1, β2, and integrin-linked kinase (Figure 7A). In agreement with these data, integrin gene expression was significantly elevated in OVX mice compared to sham controls (Figure 7B). OVX effect was present in both WT and KO mice fed WD as well as WD/alcohol. For some genes such as *Itgax* OVX, the effect was greater in WDA-fed *Prmt6* KO. We tested whether mRNA changes translated into protein changes (Figure 7D). We found that integrin α4 protein expression is undetectable in WD control livers and slightly increased in WD/alcohol mice. In contrast, ITGA4 expression was greatly elevated in OVX livers independent of the diet (Figure 7D).

To test if this mechanism is relevant in human disease, we analyzed integrin expression (*ITGA4, ITGAX*) in human blood monocytes from patients with liver disease (Figure 7E) obtained from KUMC Liver Bank. We found that monocytes from female patients with ALD had lower levels of integrin gene expression compared to male patients. Interestingly, this difference was reversed in patients with HCV.

### Female sex hormones protect from alcohol and WD-induced fibrosis and inflammation via integrin signaling

**FIGURE 8:**
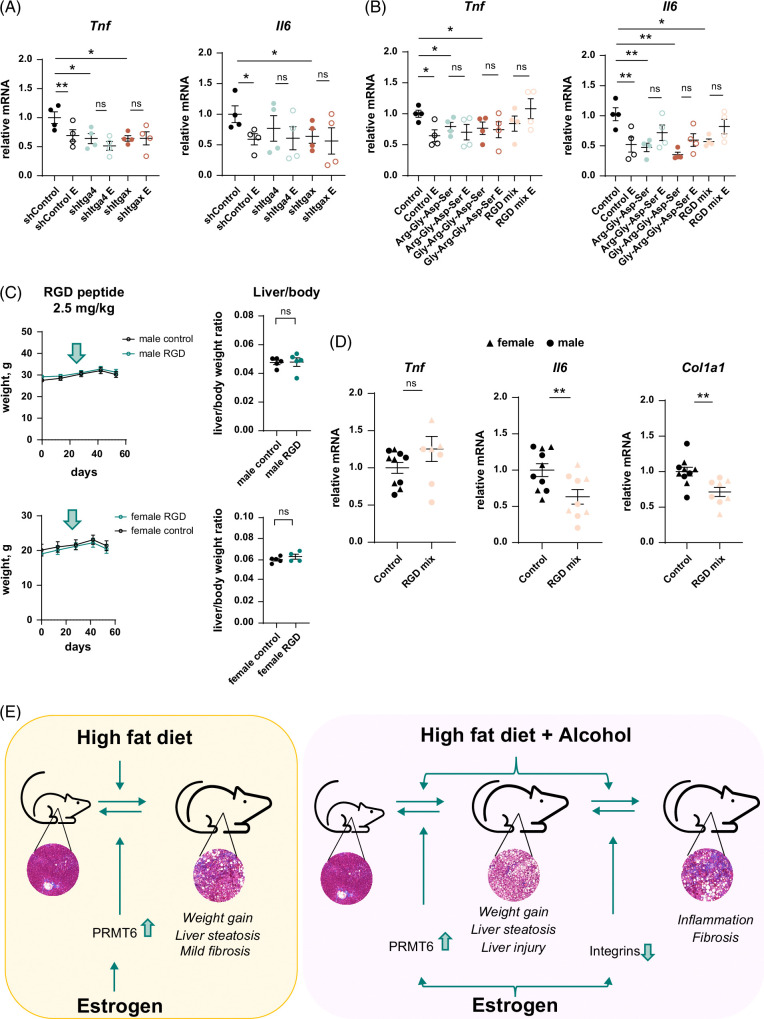
Estrogen suppresses proinflammatory and profibrotic gene expression via integrins. (A) Relative gene expression in mouse peritoneal macrophages transfected with shRNA specific for *Itga4*, *Itgax*, or control shRNA treated in vitro with 100 nM of 17β-estradiol solution (E). N=4 independent experiments. *, *p*<0.05, **, *p*<0.01 by paired *t* test. (B) Relative gene expression in mouse peritoneal macrophages treated *in vitro* with 100 nM of 17β-estradiol solution (E) in the presence or absence of 10 µg/mL of indicated RGD peptides individually or combined. N=4 independent experiments. *, *p*<0.05, **, *p*<0.01 by paired *t* test. (C and D) Male and female mice were fed WD with alcohol in the drinking water for 8 weeks (20% alcohol). Mice received twice weekly 2.5 mg/kg RGD peptide mix during last 4 weeks of feeding. (C) Weight changes over time. Arrow indicates the start of the treatment. Right. Liver-to-body weight ratios. (D) Whole liver mRNA. N=9–10 mice per group in male (●) and female (▲) mice. **, *p*<0.01 by student *t* test. (E) Summary of the presented results. Abbreviations: PRMT6, protein arginine methyltransferase 6; RGD, Arg-Gly-Asp; shRNA, small hairpin RNA.

To evaluate the role of estrogen-mediated suppression of integrin signaling, we tested the role of integrin signaling using small hairpin RNA-mediated knockdown in isolated macrophages (Figure 8A). We found that estrogen treatment significantly reduced gene expression of proinflammatory cytokines such as *Tnf* and *Il6*. Integrin gene (*Itga4* or *Itgax*) knockdown similarly reduced *Tnf* and *Il6* gene expression, and estrogen treatment had no additional effect in the knockdown cells, suggesting that reduced proinflammatory gene expression in estrogen-treated macrophages is mediated by estrogen-mediated integrin suppression. We next tested whether we could mimic these effects using peptides containing Arg-Gly-Asp integrin-binding motif (Figure 8B). In vitro treatment with 2 Arg-Gly-Asp-containing peptides phenocopied the effect of integrin knockdown, peptides reduced proinflammatory gene expression, and abolished the effect of estrogen on macrophages (Figure 8B).

Finally, we tested the effect of integrin inhibition *in vivo*. Male and female mice were fed WD with alcohol in the drinking water (20% alcohol for 8 weeks) and received twice weekly injections of Arg-Gly-Asp peptide mix (2.5 mg/kg each) during the last 4 weeks of feeding. Peptide injections did not affect weight gain or liver-to-body weight ratios (Figure 8C). We found that peptide-treated mice had significantly lower levels of *Col1a1* and *Il6* mRNA, suggesting that inflammation and fibrosis were attenuated in these mice (Figure 8D).

## DISCUSSION

Alcohol-associated liver disease is a complex disease influenced by both environmental and genetic factors, such as age and sex. Sex is an important variable; for decades, it has been known that the consequences of alcohol consumption are different in males and females.^25–29^ Several studies indicated that alcohol metabolism contributed to sex differences.^28–30^ Recent single-cell RNA-seq studies confirmed that alcohol effects on every liver cell population were indeed sex specific.^31,32^ In human patients, females have higher mortality in acute alcohol-associated hepatitis, even though men have higher serum creatinine, ALT, and GGT concentrations.^25^ Estrogen is considered to contribute to increased susceptibility of women to alcohol-associated liver disease.^33,34^ This hypothesis is supported mostly by in vitro studies in isolated cells. On the other hand, the role of estrogen in metabolic dysfunction–associated steatotic liver disease (MASLD) progression is considered mostly protective. Recent rodent studies revealed that males’ and females’ steatotic liver disease progression differ in innate and adaptive immunity, fibrosis signaling, and other pathways that involve multiple nonparenchymal cell population populations.^20,27,35–37^ Rodent gonadectomy experiments in mice suggested that these pathways are, in part, regulated by sex hormone signaling.^35^ In humans, lack of estrogen in postmenopausal women is associated with increased susceptibility to MASLD.^1,2^ This notion is supported by multiple studies in rodent models of liver disease.

The combination of MASLD with heavy alcohol consumption is termed MetALD (MASLD + alcohol 140–350 g/wk and 210–420 g/week for females and males, respectively). Recent clinical studies examining existing databases such as UK Biobank or National Health and Nutrition Examination Survey (NHANES) data set using this new nomenclature revealed that the MetALD (MASLD with heavy alcohol consumption) group comprises predominantly males (66% in UK Biobank or 71% in NHANES vs. 55%–60% in MASLD controls) and have higher serum ALT and aspartate aminotransferase levels but lower incidence of diabetes and dyslipidemia compared to MASLD.^38,39^ However, no systematic assessment of the role of female sex and female sex hormone signaling in MetALD was performed. In this study, we examined the role of female sex hormones and arginine methyltransferase PRMT6 in liver disease development using a combination of high-fat diet (WD) feeding and alcohol in the drinking water.

We found that female sex hormones protected mice from WD/alcohol-induced liver disease by promoting PRMT6 expression, which prevented WD-induced weight gain and liver steatosis, and by suppressing integrin gene expression, which protected female mice from alcohol-induced liver fibrosis (Figure 8E). Loss of female sex hormone signaling resulted in increased weight gain, steatosis, and fibrosis in mice fed WD/alcohol. Interestingly, in control mice fed WD with plain water, loss of female sex hormone signaling also resulted in weight gain, liver steatosis, and mild fibrosis; however, these were less pronounced and fully dependent on PRMT6 (Figure 8E).

Previously, we identified PRMT6 as a key regulator of liver fibrosis development in mice fed high-fat diet, alcohol, or treated with thioacetamide as well as mice on a chow diet at 1 year of age.^18^ One of the main protective functions of PRMT6 is macrophage integrin methylation, which reduces their profibrotic function in liver disease induced by multiple factors: high-fat diet, alcohol, thioacetamide treatment, or aging.^23^ In this study, we found that female sex hormone signaling and specifically, estrogen, suppresses integrin gene expression.

Thus, PRMT6 function in protecting female mice from alcohol-induced liver fibrosis is reduced, which correlates with observed minor differences in fibrosis levels between female wild type and *Prmt6* knockout mice fed alcohol. In contrast, the effect of alcohol and *Prmt6* knockout is synergistic in male mice. We demonstrated that in agreement with this hypothesis, estrogen treatment in male mice protected them from liver fibrosis and reduced differences between wild-type and knockout mice.

Our study revealed a complex interplay between female hormone signaling and PRMT6 activity, which is especially evident from our proteomic study. An intriguing finding from that study was that PRMT6 loss and female hormone signaling loss promoted similar changes in liver proteome; in contrast, the combination of both revealed many novel targets of female sex hormone signaling in the liver. Several of these targets were involved in cellular adhesion and integrin signaling. Our data suggests that estrogen-mediated integrin regulation is important factor of ALD development.

Previous studies indicated that PRMT6 has very narrow substrate specificity and methylates very few targets outside the nucleus.^40^ Despite the small number of substrates, it regulates multiple processes in the liver, including lipid metabolism, inflammation, and fibrosis development. It is an important regulator in humans as well. Genetic polymorphisms in *PRMT6* gene were found to be associated with body weight, cholesterol, and bilirubin levels, as well as serum alanine aminotransferase measurement, suggesting that PRMT6 in humans could be involved in liver function. Previously, we identified that liver PRMT6 levels negatively correlate with liver fibrosis in patients with liver disease.^23^ Here, we found that PRMT6 regulates weight gain and liver steatosis, while its effect on fibrosis is sex dependent and mediated by estrogen signaling. Further studies are necessary to test the role of *PRMT6* genetic polymorphisms in the susceptibility to liver disease (ALD and/or MetALD) and the role of estrogen, especially in premenopausal and postmenopausal female patients.

Interestingly, PRMT6 genetic polymorphisms were associated in humans with testosterone levels and sex hormone–binding globulin levels in postmenopausal women. This is another aspect that could contribute to sex differences observed in our study and a subject of a future investigation.

One of the limitations of our study is the use of whole-body knockout mice. PRMT6 is primarily expressed in nonparenchymal cells, and its expression in liver macrophages is the highest compared to stellate cells or endothelial cells, 2 other main non-parenchymal cells populations in the liver. It is likely, however, that the weight gain effect reported here could be mediated by extrahepatic PRMT6, and the effect on liver steatosis could be due to PRMT6 expression in hepatocytes. Previously, we have shown that PRMT6 and integrin signaling in liver macrophages is mediating the effects on liver fibrosis development.^23^ However, integrin-dependent mechanisms could be important in other cell types as well. While integrins α4 and αx are enriched in macrophages. Integrin β1 is expressed in multiple cell types, and it is an essential regulator of liver disease progression via its roles in stellate cells, hepatocytes, and endothelial cells.^41–45^ Further studies using cell type–specific knockout mice are necessary to define the role of specific integrins in estrogen-mediated protective effect for LD development.

In conclusion, we showed that female sex hormone signaling and PRMT6 interplay is an important modulator of ALD development through regulation of liver steatosis, liver injury, and fibrosis in the setting of high-fat diet feeding, which has important implications for patients with MetALD. Specifically, we found that estrogen increased PRMT6 levels and suppressed liver integrin gene expression and thus prevented fibrosis development. Several integrin target therapies are now in clinical trials for different inflammatory conditions and cancer. Our data suggest that future potential integrin-targeted therapy for ALD must consider estrogen and PRMT6 levels (or polymorphism status) to define the candidates who might benefit the most.
